# The Association between Oxidative Balance Score and Urinary Levels of 8-Hydroxydeoxyguanosine among Japanese Adults

**DOI:** 10.3390/nu15214533

**Published:** 2023-10-26

**Authors:** Hinako Nanri, Megumi Hara, Yuichiro Nishida, Chisato Shimanoe, Yun-Shan Li, Hiroshi Kasai, Kazuaki Kawai, Yasuki Higaki, Keitaro Tanaka

**Affiliations:** 1 Department of Physical Activity Research, National Institutes of Biomedical Innovation, Health, and Nutrition, Osaka 566-0002, Japan; 2 Laboratory of Gut Microbiome for Health, Microbial Research Center for Health and Medicine, National Institutes of Biomedical Innovation, Health, and Nutrition, Osaka 567-0085, Japan; 3 Department of Preventive Medicine, Faculty of Medicine, Saga University, Saga 849-8501, Japan; harameg@cc.saga-u.ac.jp (M.H.); ynishida@cc.saga-u.ac.jp (Y.N.); tanakake@cc.saga-u.ac.jp (K.T.); 4 Department of Pharmacy, Saga University Hospital, Saga 849-8501, Japan; chisatos@cc.saga-u.ac.jp; 5 Department of Environmental Oncology, Institute of Industrial Ecological Sciences, University of Occupational and Environmental Health, Kitakyushu 807-8555, Japan; liyunsha@med.uoeh-u.ac.jp (Y.-S.L.); h-kasai@med.uoeh-u.ac.jp (H.K.);; 6 Laboratory of Exercise Physiology, Faculty of Sports and Health Science, Fukuoka University, Fukuoka 814-0180, Japan; higaki@fukuoka-u.ac.jp

**Keywords:** oxidative balance score, antioxidant, urinary 8-OHdG, Japanese

## Abstract

The oxidative balance score (OBS), wherein higher OBSs indicate lower oxidative stress, was designed to assess a composite measure of multiple pro-oxidant and antioxidant effects on an individual’s oxidative stress status. This study aimed to evaluate whether OBSs were inversely associated with urinary levels of 8-hydroxydeoxyguanosine (8-OHdG)–an oxidative stress marker–among Japanese adults. This cross-sectional study was based on data obtained during 2010–2012. Overall, 7552 participants from the J-MICC Study Saga who answered a self-administered food frequency questionnaire were recruited for the final analysis. OBSs were calculated from 11 pro-oxidant and antioxidant lifestyle factors, including dietary intake, physical activity, and alcohol and smoking status. Urinary 8-OHdG values were corrected by creatinine level (ng/mg creatinine). Our findings revealed a higher total OBS was significantly associated with lower urinary 8-OHdG/creatinine levels after adjustment for covariates in men and women (*p* for trend < 0.01 in both sexes). We performed a multiple regression analysis of the association between OBSs and urinary 8-OHdG/creatinine levels stratified by age, body mass index (BMI), and menopausal status and found consistent negative associations in most groups for both sexes. No significant differences in the 60–64 age group for women (standardized *β* = −0.09, *p* = 0.13) or BMI < 18.5 kg/m^2^ for men (standardized *β* = −0.18, *p* = 0.17) were observed. A higher OBS had a strong inverse association with urinary 8-OHdG/creatinine levels in men and women among Japanese adults. The OBS might be a useful tool for evaluating the roles of oxidative stress-related lifestyle factors, including diet.

## 1. Introduction

Oxidative stress, defined as the disruption of the balance between pro-oxidants and antioxidants [1], has been implicated in the etiology and pathophysiology of several chronic diseases, which in turn act as leading contributors to mortality [1,2]. Oxidative stress is influenced by intrinsic factors, such as oxidative phosphorylation [3], intracellular antioxidant enzyme activity [4], macromolecular damage [5], and extrinsic factors. It has been suggested that smoking [6], alcohol consumption [7], and iron excess [8] may act as exogenous oxidative enhancers and increase oxidative stress. In contrast, antioxidant factors, such as vitamin C and carotene, which are abundant in fruits and vegetables, can counteract or reduce the effects of reactive oxygen species, thereby reducing oxidative stress [9]. Additionally, high physical activity was inversely associated with low levels of urinary 8-hydroxydeoxyguanosine (8-OHdG) [10], a commonly used marker of DNA oxidative stress [11,12].

Since our diet consists of a combination of foods containing multiple and various nutrients, it is believed that their effects may be enhanced or counteracted in the body [13,14]. Recent studies on diet and health have demonstrated that combinations of multiple antioxidant factors are more strongly associated with disease risk than when individual nutrients are considered [15,16]. It has also been suggested that a combined measure that takes into account multiple lifestyle factors associated with oxidative stress might be a more accurate indicator of health outcomes than individual pro-oxidant or antioxidant factors [17,18].

The oxidative balance score (OBS) was developed to more accurately assess the combined pro-oxidant and antioxidant exposure status using dietary and non-dietary factors (e.g., alcohol consumption and smoking) [19,20], and various versions of the OBS have been used to assess health outcomes [20,21,22,23,24]. In general, antioxidant factors contribute positively, whereas pro-oxidant factors contribute negatively; therefore, a higher OBS reflects a predominance of antioxidant exposures over pro-oxidant exposures. Some studies have focused on the relationship between OBSs and oxidative stress biomarkers [21,22,24,25,26,27]; however, most studies have been conducted in the United States, and the number of reports is still limited. In Asian countries, only one study [26] was conducted in healthy Koreans, in which the OBS component factors were limited to a relatively small number, including dietary fat, iron, and vitamin C. In addition, there remains a debate about the constituent factors of the OBS. Although vitamin D and folic acid have been reported to have strong antioxidant properties [28,29], there has been little discussion about whether these factors should be included as components of the OBS in most published studies on the association between various exposures and the OBS [30]. Moreover, in most previous studies of the OBS, although total polyunsaturated fatty acids (PUFAs) have been considered as pro-oxidant factors, *n*-3 PUFAs have been shown to have anti-inflammatory effects [30]. Therefore, it is necessary to distinguish between *n*-3 and *n*-6 PUFAs and whether these factors should be included as pro-oxidant or antioxidant factors in the OBS.

This study aimed to examine whether the OBS was inversely associated with urinary levels of 8-OHdG as an oxidative stress marker in a large Japanese population. Although we used a multivariate analysis to examine the association between the OBS and 8-OHdG after adjusting for the effects of age, body mass index (BMI), and menopausal status, this might not cover the differences within strata for these variables. Therefore, we have also performed the analysis by the stratification of these factors.

## 2. Subjects and Methods

### 2.1. Study Subjects

The baseline survey of the Japan Multi-Institutional Collaborative Cohort Study in the Saga region (designated the Saga J-MICC Study) was conducted from 2005 through 2007 in Saga, Japan [31]. A total of 61,447 registered residents between the ages of 40 and 69 years were invited by mail to participate in the baseline survey, 12,078 of whom ultimately agreed to participate. After excluding participants who moved out of the area (*n* = 423), died (*n* = 163), and withdrew from the study (*n* = 9) during follow-up, 11,483 people were again invited to participate in a face-to-face second survey between 2010 and 2012, 5 years after the baseline survey. A total of 8454 subjects aged 45–74 years participated in the second survey [32], and the current study was undertaken on these second-survey participants. Written informed consent was obtained from each participant after explaining the purpose, content, and conditions of the study. The research protocol was approved by the ethics committees of the Saga University Graduate School of Medicine (approval No. 17-11), Nagoya University Graduate School of Medicine (approval No. 253), and National Institute of Biomedical Innovation, Health and Nutrition (NIBIOHN-135-01).

From the 8454 participants, 902 were excluded for the following reasons: (i) missing data on urinary 8-OHdG and creatinine levels (*n* = 49); (ii) missing data on individual factor components of the OBS (*n* = 159); (iii) any history of a possible inflammation-related disease (cardiovascular disease, cancer, liver disease, or chronic renal failure; *n* = 687); and (iv) extremely low or high dietary energy intake (*n* = 7), that is, dietary energy intake <800 or ≥4500 kcal/day for men and <500 or ≥3500 kcal/day for women, because such intakes are unrealistic and hence of questionable validity. Finally, there were 7552 eligible participants (3083 men and 4469 women).

### 2.2. Data Collection

Height and weight were measured to the nearest 0.1 cm and 0.1 kg, respectively, and BMI was calculated. A self-administered questionnaire was used to ascertain smoking status, dietary habits, current medication, and disease history. Regarding smoking habits, subjects were first asked about their current smoking status (and cessation time for former smokers). Current and former smokers reported their usual cigarette consumption (cigarettes per day) and the age at which they started smoking. Physical activity level (PAL) was assessed using a single-axis accelerometer (Kenz Lifecorder EX; Suzuken Co., Ltd., Nagoya, Japan) on either side of the hip, except when sleeping or bathing, for 10 days. PAL was calculated by dividing the total energy expenditure (kcal/day) by the basal metabolic rate (kcal/day). The former was estimated from the accelerometer as the average daily (excluding the first 3 days) energy expenditure, and the latter was defined as basal metabolic standard [33] × body surface area [34] × 24 h.

### 2.3. Urine Sampling and Laboratory Assay

Participants were asked to provide an 8 mL urine sample immediately after waking. We collected 8405 urine samples from 8454 participants. After using approximately 3 mL to measure density, pH, and levels of protein, bilirubin, ketone, occult blood, and sugar, the remaining urine was transferred to Eppendorf tubes (1.5 mL × 3) and stored at −80 °C until needed. Levels of urinary 8-OHdG and creatinine were measured at the Department of Environmental Oncology, Institute of Industrial Ecological Sciences, University of Occupational and Environmental Health and OHG Institute Co., Ltd. (Kitakyushu, Japan) by an established method using a high-performance liquid chromatography system [12]. The accuracy of the measurement, which was estimated based on the recovery of an added 8-OHdG standard, was reported to be 90% to 98%, and the coefficient of variation was reported to be ±7% using this method [35]. The limit of detection of this assay was 0.5 ng/mL.

### 2.4. Blood Analysis

Venous blood was drawn from each participant, and serum, plasma, and buffy coat were separated within 3 h and stored at −80 °C until testing. Parts of the stored serum specimens were sent to an external laboratory (SRL, Hachioji, Japan), and serum ferritin levels were measured using a latex-enhanced immunonephelometric assay on a BN II analyzer (Dade Behring, Marburg, Germany).

### 2.5. Dietary Survey

Dietary information was collected using a validated self-administered food frequency questionnaire (FFQ) [36,37,38,39]. In this study, the dietary intake of major nutrients involved in oxidative stress, namely, carotene, vitamin E, vitamin C, saturated fatty acid (SFA), *n*-3 PUFA, and *n*-6 PUFA, was assessed at the 5-year follow-up survey. In the FFQ, we asked participants to report their frequency of intake of 47 food and beverage items over the past year to assess the average intake. Daily nutrition intake was then calculated using a program developed at the Department of Public Health, Nagoya City University School of Medicine, based on the standard tables of food consumption in Japan (5th revision) [40]. For the item that inquired about alcohol consumption, the frequency and quantity of six different alcoholic drinks were assessed. The total ethanol consumption per day for current drinkers was estimated based on beverage-specific ethanol concentrations. Fatty acid and vitamin intake from supplements were not included in the exposures. All nutritional covariates except alcohol were adjusted for total energy intake using the density method [41].

### 2.6. Calculation of the OBS

The OBS was calculated by combining information from a total of 11 a priori selected pro-oxidant and antioxidant factors, including dietary intake of carotene, vitamin E, vitamin C, serum ferritin, *n*-3 and *n*-6 PUFA, SFA, PAL, alcohol consumption, smoking status, and regular use of non-steroidal anti-inflammatory drugs (NSAIDs) (Supplementary Table S1). The continuous variables reflecting pro-oxidant (serum ferritin, *n*-3 and *n*-6 PUFA, and SFA) and antioxidant (carotene, vitamin E, vitamin C, and PAL) exposures were divided into low, medium, and high categories based on tertile values for each exposure. For antioxidants, the first through third tertiles were assigned 0–2 points, whereas the corresponding point assignment for pro-oxidants was the reverse (0 points for the highest tertile and 2 points for the lowest tertile). A similar scoring approach was used for pro-oxidant and antioxidant categorical variables. For alcohol consumption, nondrinkers, moderate drinkers (>0–<23 g of ethanol/day), and heavy drinkers (≥23 g of ethanol/day) received 2, 1, and 0 points, respectively. Smoking status was categorized as never smoker (2 points), former smoker (1 point), and current smoker (0 points). For NSAID use, 0 points were assigned to participants with no regular use and 2 points to those with regular use. The overall OBS was then calculated by adding up the points assigned to each participant, with a higher OBS representing the predominance of antioxidant over pro-oxidant exposures.

### 2.7. Statistical Analysis

Characteristics were compared between men and women using the *t* test (continuous variables) and chi-square test (categorical variables). All analyses were performed separately for men and women since the number of subject characteristics was found to differ between the sexes (Supplementary Table S2). Serum ferritin, urinary 8-OHdG, and serum creatinine levels were natural log-transformed prior to analysis to obtain a better approximation to the normal distribution. Given that this study evaluated 8-OHdG using spot urine samples, the concentration of urinary 8-OHdG (ng/mL) was divided by the creatinine level (mg/mL), which serves as an indicator of urine dilution [42]. To determine the association between the OBS scores and urinary 8-OHdG/creatinine levels, a multiple linear regression analysis was performed. The selected characteristics of participants across the OBS quartiles were evaluated using the linear regression analysis for continuous variables and the Mantel–Haenszel test for categorical variables. Adjusted geometric means of urinary 8-OHdG/creatinine levels and their 95% confidence intervals (CIs) by quartiles of the OBS were computed using the general linear model procedure of SAS (analysis of covariance) in three different models. The first model was not adjusted. The second model was adjusted for age (years, continuous variable), total energy intake (kcal, continuous variable), hypertension (yes or no, categorical variable), diabetes mellitus (yes or no, categorical variable), dyslipidemia (yes or no, categorical variable), and menopausal status (in women only, categorical variable). In the third model, the BMI was additionally controlled. The multivariable-adjusted odds ratios (ORs) (95% CIs) for urinary 8-OHdG/creatinine levels according to the dietary (carotene, vitamin E, vitamin C, *n*-3 PUFA, *n*-6 PUFA, SFA, and serum ferritin levels) and lifestyle OBS (PAL, use of NSAIDs, alcohol consumption, and smoking status) were also obtained to assess the standardized *β* coefficients of the dietary and lifestyle OBS to urinary 8-OHdG/creatinine levels. Moreover, analyses were further stratified by the age group (aged 45–49, 50–59, 60–64, 65–69, and 70–74 years), BMI (<18.5, 18.5–24.9, and ≥25.0 kg/m^2^), and menopausal status (pre- and postmenopausal). All statistical analyses were performed using the SAS statistical software package (Ver. 9.4; SAS Institute, Cary, NC, USA).

## 3. Results

The OBS ranged from 3 to 18 for men and 2 to 19 for women, and the mean scores (standard deviation) were 10.0 (2.5) and 11.5 (2.4), respectively. Table 1 shows the results of the multiple regression analysis to assess the association between individual factor components of the OBS, total OBS, OBS from dietary factors, and OBS from lifestyle factors and urinary 8-OHdG/creatinine. Serum ferritin levels, alcohol consumption, smoking status, and vitamin C levels (only in men) were significantly associated with urinary 8-OHdG/creatinine levels; the standardized *β* values were 0.50, −0.08, 0.10, and −0.05 for men and 0.64, −0.04, and 0.05 for women, respectively (*p* < 0.01 for all). We did not observe a significant association between other dietary factors (PAL, use of NSAIDs, and urinary 8-OHdG/creatinine levels). There was a significant association between the total OBS (standardized *β* = −0.16, *p* < 0.01 for men, −0.13, *p* < 0.01 for women), dietary OBS (standardized *β* = −0.15, *p* < 0.01 for men, −0.13, *p* < 0.01 for women), lifestyle OBS, and urinary 8-OHdG/creatinine levels (standardized *β* = −0.09, *p* < 0.01 for men, −0.05, *p* < 0.01 for women). In addition, to examine whether vitamin D, folic acid, and *n*-PUFA should be included as antioxidant factors, we performed a multiple regression analysis to assess the association between the OBS and urinary 8-OHdG/creatinine levels, including these factors (Supplementary Table S3). When the OBS included vitamin D and folic acid intake, the standardized *β* was smaller than that obtained when the OBS was constructed without these factors. The same result was observed when the OBS was constructed using *n*-3 PUFA as a component of the antioxidant factors.

Table 2 shows the characteristics of the study participants according to the OBS quartiles. Participants with a higher OBS tended to be older (men only), had a lower BMI and total energy intake (women only), and had a history of dyslipidemia (men only). The relationship between the OBS and its components was similar for both sexes; participants with the highest OBS tended to have a higher intake of antioxidants, higher PAL, and a higher proportion of antipyretic analgesic use than those with the lowest OBS. In contrast, they had a lower intake of fatty acids, a lower proportion of smokers and drinkers, and lower serum ferritin levels.

Table 3 shows the adjusted geometric means of urinary 8-OHdG/creatinine levels and their 95% CI according to the quartiles of the OBS in men and women. The total OBS was significantly associated with urinary 8-OHdG/creatinine levels in men and women before and after adjustment for all covariates (*p* for trend <0.01 all). Similar results were obtained for the association between OBS equal interval categories and urinary 8-OHdG/creatinine levels (Supplementary Table S4).

Furthermore, we performed a multiple regression analysis of the association between the OBS and urinary 8-OHdG/creatinine levels stratified by age, BMI, and menopausal status (in women only) (Table 4). Consistent negative associations were observed both in men and women, but it was not significant in the 60–64 age group for women (standardized *β* = −0.09, *p* = 0.13) and BMI < 18.5 kg/m^2^ for men (standardized *β* = −0.18, *p* = 0.16).

## 4. Conclusions

In our cross-sectional study, we found a negative association between the OBS and urinary 8-OHdG/creatinine levels in the J-MICC Saga participants. These results were consistent in almost all subgroups of age or BMI in both sexes. Additionally, similar results were obtained in the subgroup of women with menopausal status. Our results suggest that the OBS constructed using a combination of questionnaires and biomarkers is applicable to the Japanese population.

Similar results have been observed in previous studies using OBSs developed using either questionnaire-based methods [21,26] or a combination of questionnaires and biomarkers [22,24,25,27]. A case–control study of colorectal adenoma by Dash et al. [21] reported that questionnaire-derived OBSs were significantly associated with F_2_-isoprostanes (FIP) levels as a useful biomarker of oxidative stress. Two case–control studies [22,24] and one cross-sectional study [27] also found that those in the lowest category of the combined questionnaire and biomarker-derived OBS had significantly lower levels of FIP compared with those in the highest category. Annor et al. [25] developed a total OBS that assessed dietary antioxidant and pro-oxidant status by biomarker components; a negative correlation between the OBS and FIP level was observed. The OBS constructed within a Korean study population by Cho et al. [26] included fewer components than most of those described above, and a higher OBS had a strong inverse association with gamma-glutamyl transferase levels (GGT) as a biomarker of oxidative stress. Interestingly, no statistically significant correlations were identified between individual factors, such as β-carotene, vitamin C, physical activity, and the use of nonsteroidal anti-inflammatory drugs as antioxidants, and GGT levels. In the present study, we have seen a significant association between the OBS and another oxidative stress marker (i.e., 8-OHdG) in a larger Asian population, and we believe this is the first study to evaluate the association between the OBS and a urinary biomarker of oxidative stress in a Japanese population.

In our study, serum ferritin as a pro-oxidant was strongly associated with urinary 8-OHdG/creatinine levels; high body iron levels could catalyze oxidative reactions, promoting iron-induced lipid and protein peroxidation [8]. Furthermore, the influence of serum ferritin on oxidative stress may involve complex biological interactions, encompassing factors within the OBS. For instance, vitamin C is known to enhance the absorption of dietary iron [43]. In addition, nutrients with antioxidant properties have been reported to have higher biological availability in the presence of risk factors that elevate oxidative stress [44,45]. Therefore, it may be important to incorporate serum ferritin into the OBS to investigate its association with oxidative stress and disease. Most previous studies have used dietary iron as a component of the OBS [21,24,26,27]; however, in this study, the use of serum ferritin as a component of the OBS was more strongly associated with urinary 8-OHdG than the use of dietary iron. Obesity has also been reported to be independently associated with increased oxidative stress markers [46], which may mediate and confound the association of the OBS with health outcomes, since obesity is caused by individual lifestyle habits such as diet, smoking, and alcohol consumption [47]. Therefore, we treated BMI as an adjustment factor and not as an inclusion factor in the OBS.

Although high dietary fat has the highest pro-oxidant potential [30], it remains unclear whether PUFAs should be considered as antioxidant or pro-oxidant factors. The mechanism of action of *n*-3 PUFAs, such as eicosapentaenoic acid and docosahexaenoic acid, is still not fully understood, but they have been shown to have anti-inflammatory effects [48]. In contrast, a higher intake of *n*-6 PUFAS (e.g., gamma-linolenic acid) has been associated with increased oxidative stress through increased free-radical production [49]. Our results showed that the standardized *β* in the association between the OBS and urinary 8-OHdG/creatinine level was smaller when *n*-3 PUFA was considered as an antioxidant component than when it was considered as a pro-oxidant component. Moreover, other vitamins with antioxidant properties, such as folic acid and vitamin D, have not been considered in most OBSs. Only two previous studies on the OBS included vitamin D as an antioxidant [50,51]. However, our results showed that the standardized *β* between the OBS (constructed with vitamin D and folic acid) and urinary 8-OHdG level had a smaller association than the OBS constructed without these factors.

Our study had some limitations. Since the study had a cross-sectional design, it was not possible to infer a temporal relationship between the OBS and 8-OHdG/creatinine levels. We excluded participants with a history of cardiovascular disease, cancer, liver disease, or chronic renal failure, which could lead to dietary or lifestyle changes. We also performed an analysis that excluded participants with high serum ferritin levels (>1000 ng/mL, *n* = 5), but this did not alter the results. However, this study design continues to limit the ability to establish causal relationships. We used self-reported intakes to assess pro-oxidant and antioxidant exposures; thus, misclassifications might have occurred. Misclassifications would have weakened the association shown in the current study and biased the results toward the null hypothesis. It has long been acknowledged that dietary questionnaires may not capture all the possible sources of each nutrient. However, the validity and reliability of the FFQ used in our study have been extensively evaluated [36,37,38,39], and any misclassification is expected to be nondifferential. It has also been suggested that the bioavailability of antioxidant nutrients varies according to the form of food (food/drink), but it is not possible to consider the type of food from which the antioxidant is taken. In this study, participants were not randomly selected, and thus may have exhibited a higher level of health consciousness, thereby creating a possibility for selection bias. These limitations may hamper the generalizability of the results.

In conclusion, the results from this large cross-sectional study demonstrated that a higher OBS was associated with urinary 8-OHdG/creatinine levels in men and women. Moreover, these associations were also observed in almost all groups of age, BMI, and menopausal status. These findings confirm the results of previous studies and suggest that the OBS might be a useful tool for evaluating the roles of oxidative stress-related lifestyle factors, including diet. Further prospective studies are needed to confirm these associations.

## Figures and Tables

**Table 1:** Multiple regression analysis between OBS component individual factors or total OBS and log-transformed urinary 8-OHdG/creatinine level in Japanese men and women.

	Men (*n* = 3083) ^a^				Women (*n* = 4469) ^b^			
	Standardized *β*	*β*	95% CI	*p*	Standardized *β*	*β*	95% CI	*p*
*Antioxidant*								
Carotene (μg/1000 kcal)	0.02	0.00002	(−0.00002, 0.00005)	0.39	0.01	0.000005	(−0.00001, 0.00002)	0.61
Vitamin E (mg/1000 kcal)	−0.05	0.020	(−0.06, 0.02)	0.29	−0.04	−0.015	(−0.04, 0.005)	0.14
Vitamin C (mg/1000 kcal)	0.05	0.0006	(0.0002, 0.003)	0.02	0.02	0.0005	(−0.0001, 0.001)	0.11
Physical activity level	−0.01	0.075	(−0.21, 0.08)	0.41	−0.02	−0.122	(−0.26, 0.02)	0.09
Use of NSAIDs	0.01	0.031	(−0.04, 0.08)	0.52	0.01	0.019	(−0.03, 0.07)	0.46
*Pro*-*oxidant*								
*n*-3 PUFA (g/1000 kcal)	−0.002	0.00004	(−0.00008, 0.00007)	0.94	0.002	0.000002	(−0.00005, 0.00006)	0.93
*n*-6 PUFA (g/1000 kcal)	0.01	0.00001	(−0.00002, 0.00002)	0.78	0.02	0.000005	(−0.00001, 0.00002)	0.41
SFA (g/1000 kcal)	0.02	0.006	(−0.007, 0.018)	0.38	0.01	0.004	(−0.004, 0.01)	0.30
Serum ferritin level (μg/L)	0.50	0.269	(0.25, 0.29)	<0.01	0.64	0.323	(0.31, 0.33)	<0.01
Alcohol consumption	−0.08	−0.028	(−0.04, −0.02)	<0.01	−0.04	−0.020	(−0.004, −0.012)	<0.01
Smoking status	0.10	0.047	(0.03, 0.06)	<0.01	0.05	0.047	(0.02, 0.07)	<0.01
Total OBS	−0.16	−0.031	(−0.04, −0.02)	<0.01	−0.13	−0.028	(−0.03, −0.02)	<0.01
Dietary OBS ^c^	−0.15	−0.039	(−0.05, −0.03)	<0.01	−0.13	−0.033	(−0.04, −0.03)	<0.01
Lifestyle OBS ^d^	−0.09	−0.028	(−0.04, −0.02)	<0.01	−0.05	−0.021	(−0.03, −0.01)	<0.01

OBS: oxidative balance score; 8-OHdG: 8-hydroxydeoxyguanosine; Standardized *β*: standardized regression coefficient; *β*: regression coefficient; CI: confidence interval; NSAID: non-steroidal anti-inflammatory drug; PUFA: polyunsaturated fatty acids; SFA: saturated fatty acids. ^a^ Adjusted for age (years, continuous), total energy intake (kcal, continuous), body mass index (kg/m^2^, continuous), hypertension (category), diabetes mellitus (category), and dyslipidemia (category). ^b^ Adjusted for age (years, continuous) total energy intake (kcal, continuous), hypertension (category), diabetes mellitus (category), dyslipidemia (category), menopausal status (category), and body mass index. ^c^ The dietary OBS included carotene, vitamin E, vitamin C, *n*-3 PUFA, *n*-6 PUFA, SFA, and serum ferritin. ^d^ The lifestyle OBS included physical activity level, use of NSAIDs, alcohol drinking, and smoking status.

**Table 2:** Selected characteristics and individual components of the score per OBS quartile.

	Men			Women		
	Q1 (*n* = 501)	Q4 (*n* = 818)	*p* Trend ^a^	Q1 (*n* = 872)	Q4 (*n* = 1511)	*p* Trend
Age (years)	61.2 (7.7)	62.6 (7.8)	<0.01	60.3 (8.0)	60.5 (8.3)	0.70
Body mass index	24.1 (3.2)	23.4 (2.8)	<0.01	22.5 (3.3)	22.1 (3.0)	0.02
Total energy intake (kcal/d)	1876 (319)	1897 (319)	0.06	1462 (245)	1524 (218)	<0.01
Hypertension, *n* (%)	167 (33.3)	236 (28.9)	0.17	168 (19.3)	268 (17.7)	0.37
Diabetes, *n* (%)	50 (10.0)	79 (9.7)	0.87	43 (4.9)	65 (4.3)	0.85
Dyslipidemia, *n* (%)	71 (14.2)	152 (18.6)	0.01	160 (18.4)	295 (19.5)	0.48
Carotene (μgRE/1000 kcal)	1205 (358)	1782 (695)	<0.01	1875 (625)	2788 (1045)	<0.01
Vitamin E (mg/1000 kcal)	4.0 (0.9)	4.3 (1.1)	<0.01	5.5 (1.3)	5.8 (1.4)	<0.01
Vitamin C (mg/1000 kcal)	39.7 (11.3)	56.1 (18.0)	<0.01	62.0 (18.2)	83.4 (24.8)	<0.01
*n*-3 PUFA (g/1000 kcal)	1.3 (0.3)	1.2 (0.3)	<0.01	1.6 (0.4)	1.5 (0.1)	<0.01
*n*-6 PUFA (g/1000 kcal)	5.9 (1.3)	5.5 (1.3)	<0.01	7.7 (1.9)	7.0 (1.7)	<0.01
SFA (g/1000 kcal)	5.9 (1.3)	5.6 (1.3)	<0.01	7.3 (1.6)	7.1 (1.5)	<0.01
Current smoker, *n* (%)	259 (51.7)	65 (7.8)	<0.01	138 (15.8)	10 (0.7)	<0.01
Current drinker, *n* (%)	472 (94.2)	475 (58.1)	<0.01	506 (58.0)	388 (25.7)	<0.01
Physical activity level	1.43 (0.08)	1.47 (0.11)	<0.01	1.41 (0.07)	1.47 (0.08)	<0.01
Serum ferritin level (μg/L)	157 (1.9)	62.7 (2.3)	<0.01	62.6 (2.5)	31.9 (2.8)	<0.01
Use of NSAIDs, *n* (%)	5 (1.0)	108 (13.2)	<0.01	8 (0.9)	147 (9.7)	<0.01

OBS: oxidative balance score; PUFA: polyunsaturated fatty acids; SFA: saturated fatty acids; NSAIDs: non-steroidal anti-inflammatory drugs. ^a^ Trend tests were performed by including the ordinal numbers 0 to 3 assigned to each quartile category of the OBS in a multiple linear regression analysis.

**Table 3:** Adjusted geometric means (95% CI) of urinary 8-OHdG/creatinine levels according to OBS quartiles in Japanese men and women.

		OBS								
		Q1		Q2		Q3		Q4		*p* for Trend ^a^
Men										
	*n* (%)	501 (16.2)		816 (26.4)		950 (30.8)		819 (26.5)		
	Model 1 ^b^	4.12	(3.96–4.29)	3.76	(3.65–3.88)	3.69	(3.59–3.80)	3.30	(3.20–3.41)	<0.01
	Model 2 ^c^	4.12	(3.96–4.29)	3.77	(3.65–3.89)	3.70	(3.59–3.81)	3.29	(3.19–3.40)	<0.01
	Model 3 ^d^	4.14	(3.98–4.31)	3.78	(3.66–3.90)	3.69	(3.59–3.80)	3.28	(3.17–3.38)	<0.01
Women										
	*n* (%)	873 (19.5)		1312 (29.3)		776 (17.4)		1511 (33.8)		
	Model 1	4.53	(4.38–4.68)	4.26	(4.14–4.37)	4.17	(4.02–4.32)	3.75	(3.65–3.84)	<0.01
	Model 2	4.47	(4.33–4.61)	4.25	(4.15–4.36)	4.16	(4.02–4.30)	3.78	(3.69–3.87)	<0.01
	Model 3	4.49	(4.35–4.63)	4.25	(4.02–4.29)	4.16	(4.02–4.29)	3.77	(3.69–3.86)	<0.01

OBS: oxidative balance score; CI: confidence interval; 8-OHdG: 8-hydroxydeoxyguanosine. ^a^ Trend tests were performed by including the ordinal numbers 0 to 3 assigned to each quartile category of the OBS in a multiple linear regression analysis. ^b^ Unadjusted. ^c^ Adjusted for age (years, continuous), total energy intake (kcal, continuous), hypertension (category), diabetes mellitus (category), dyslipidemia (category), and menopausal status (in women only, category). ^d^ Adjusted for age (years, continuous), total energy intake (kcal, continuous), hypertension (category), diabetes mellitus (category), dyslipidemia (category), menopausal status (in women only, category), and body mass index (kg/m^2^, continuous).

**Table 4:** Multiple regression analysis between total OBS and log-transformed urinary 8-OHdG/creatinine level, stratified by age, body mass index, and menopausal status.

	Men					Women				
	*n* (%)	Standardized *β* ^a^	*β*	95% CI	*p*	*n* (%)	Standardized *β*	*β*	95% CI	*p*
Age category, years ^a^										
45–49	314 (10.2)	−0.15	−0.031	(−0.05, −0.01)	<0.01	588 (13.2)	−0.11	−0.029	(−0.05, −0.01)	<0.01
50–54	363 (11.8)	−0.19	−0.035	(−0.05, −0.02)	<0.01	592 (13.3)	−0.16	−0.036	(−0.05, −0.02)	<0.01
55–59	504 (16.4)	−0.19	−0.035	(−0.05, −0.02)	<0.01	738 (16.5)	−0.12	−0.021	(−0.03, −0.01)	<0.01
60–64	666 (21.6)	−0.16	−0.029	(−0.01, −0.02)	<0.01	916 (20.5)	−0.09	−0.015	(−0.03, −0.004)	0.13
65–69	651 (21.1)	−0.17	−0.033	(−0.05, −0.02)	<0.01	912 (20.4)	−0.16	−0.030	(−0.04, −0.02)	<0.01
70–74	585 (19.9)	−0.16	−0.028	(−0.04, −0.01)	<0.01	723 (16.2)	−0.21	−0.043	(−0.06, −0.028)	<0.01
BMI category, kg/m^2 b^										
<18.5	74 (2.4)	−0.18	−0.033	(−0.08, 0.01)	0.16	392 (8.8)	−0.19	−0.042	(−0.06, −0.02)	<0.01
18.5–24.9	2087 (67.7)	−0.15	−0.029	(−0.04, −0.02)	<0.01	3300 (73.8)	−0.13	−0.029	(−0.04, −0.02)	<0.01
≥25.0	922 (29.9)	−0.18	−0.034	(−0.05, −0.02)	<0.001	777 (17.4)	−0.09	−0.017	(−0.03, −0.004)	<0.01
Menopausal status ^c^										
Premenopausal						809 (18.1)	−0.14	−0.034	(−0.05, −0.02)	<0.01
Postmenopausal						3660 (81.9)	−0.15	−0.027	(−0.03, −0.02)	<0.01

OBS: oxidative balance score; 8-OHdG: 8-hydroxydeoxyguanosine; Standardized *β*: standardized regression coefficient; *β*: regression coefficient; CI: confidence interval; BMI: body mass index. ^a^ Adjusted for body mass index (kg/m^2^, continuous), total energy intake (kcal, continuous), hypertension (category), diabetes mellitus (category), dyslipidemia (category), and menopausal status (in women only, category). ^b^ Adjusted for age (years, continuous), total energy intake (kcal, continuous), hypertension (category), diabetes mellitus (category), dyslipidemia (category), and menopausal status (in women only, category). ^c^ Adjusted for age (years, continuous), total energy intake (kcal, continuous), hypertension (category), diabetes mellitus (category), and dyslipidemia (category).

## Data Availability

The datasets generated and/or analyzed during the current study are available from the corresponding author on reasonable request.

## Supplementary Materials

The following supporting information can be downloaded at: https://www.mdpi.com/article/10.3390/nu15214533/s1, Table S1. OBS assignment scheme. Table S2. Selected characteristics of the study subjects per OBS quartiles. Table S3. Results of multiple regression analysis to assess the association between OBS component individual factors or total OBS and log-transformed urinary 8-OHdG/creatinine levels in Japanese men and women. Table S4. Adjusted geometric means (95% CI) of urinary 8-OHdG/creatinine levels according to equal interval categories of OBS by sex.
