# Supplementary material for: The Association between Oxidative Balance Score and Urinary Levels of 8-Hydroxydeoxyguanosine among Japanese Adults
Source: Nutrients. 2023 Oct 26;15(21):4533. doi: 10.3390/nu15214533 (PMC10647214; doi:10.3390/nu15214533)
Supplement: Supplementary file 1 [file nutrients-15-04533-s001.zip › nutrients-2688074-supplementary.pdf]

Supplemental Table S1. OBS assignment scheme

Anti-oxidant	
Carotene intake	0=low (1st tertile), 1=medium (2nd tertile), 2=high (3rd tertile)
Vitamin E intake	0=low (1st tertile), 1=medium (2nd tertile), 2=high (3rd tertile)
Vitamin C intake	0=low (1st tertile), 1=medium (2nd tertile), 2=high (3rd tertile)
Physical activity level	0=low (1st tertile), 1=medium (2nd tertile), 2=high (3rd tertile)
Regular NSAID use	0=never, 2=regular user
Pro-oxidant	
n-3 PUFA intake	2=low (1st tertile), 1=medium (2nd tertile), 0=high (3rd tertile)
n-6 PUFA intake	2=low (1st tertile), 1=medium (2nd tertile), 0=high (3rd tertile)
SFA intake	2=low (1st tertile), 1=medium (2nd tertile), 0=high (3rd tertile)
Serum ferritin	2=low (1st tertile), 1=medium (2nd tertile), 0=high (3rd tertile)
Alcohol consumption	2=nondrinker, 1=>0–<23 g of ethanol/day, 0=≥23 of ethanol/day
Smoking	2=never smoker, 1=former smoker, 0=current smoker

OBS: oxidative balance score; NSAID: non-steroidal anti-inflammatory drug; PUFA: polyunsaturated fatty acids; SFA: saturated fatty acids.

Tertiles (T) 1 through 3 include the following: carotene intakes of <1166, 1166–1628, and ≥1628 kcal for men and <1842, 1842–2553, and ≥2553 kcal for women; vitamin E intakes of <3.6, 3.6–4.5, and ≥4.5 mg/1000kcal for men and <5.0, 5.0–6.0, and ≥6.0 mg/1000 kcal for women; vitamin C intakes of <39.9, 39.9–53.5, and ≥53.5 mg/1000 kcal for men and <62.3, 62.3–79.7, and ≥79.7 mg/1000 kcal for women; physical activity level of <1.40, 1.40–1.47, and ≥1.47 for men and <1.41, 1.41–1.47, and ≥1.47 for women; n-3 PUFA intakes of <1.1, 1.1–1.3, and ≥1.3 g/1000 kcal for men and <1.4, 1.4–1.6, and ≥1.6 for women; n-6 PUFA intakes of <4.9, 4.9–6.1, and ≥6.1 g/1000 kcal for men and <6.4, 6.4–7.8, and ≥7.8 g/1000 kcal for women; SFA intakes of <5.0, 5.0–6.2, and ≥6.2 g/1000 kcal for men and <6.8, 6.8–8.0, and ≥8.0 g/1000 kcal for women; and serum ferritin of <4.33, 4.33–4.96, and ≥4.96 μg/L for men and <3.59, 3.59–4.26, and ≥4.26 μg/L for women.

Supplemental Table S2. Selected characteristics of the study subjects per OBS quartiles

	Men (n=3083)	Women (n=4469)	*P* value
Age (years)[a]	61.5 (8.0)	60.5 (8.2)	<0.01
Hypertension, n (%) [b]	902 (29.3)	837 (18.7)	<0.01
Diabetes, n (%) [b]	286 (9.3)	180 (4.0)	<0.01
Dyslipidemia, n (%) [b]	500 (16.2)	863 (19.3)	<0.01
Body mass index (kg/m^2) [a]	23.7 (2.9)	22.3 (3.2)	<0.01
Total energy intake (kcal/day) [a]	1888 (335)	1499 (229)	<0.01
Carotene (μg/1000 kcal) [a]	1512 (624)	2377 (1036)	<0.01
Vitamin E (mg/1000 kcal) [a]	4.18 (1.1)	5.66 (1.43)	<0.01
Vitamin C (mg/1000 kcal) [a]	48.5 (16.9)	74.1 (24.8)	<0.01
n-3 PUFA (g/1000 kcal) [a]	1.2 (0.3)	1.5 (3.6)	<0.01
n-6 PUFA (g/1000 kcal) [a]	5.7 (1.6)	7.4 (1.9)	<0.01
SFA (g/1000 kcal) [a]	5.8 (1.4)	7.6 (1.6)	<0.01
Physical activity level [a]	1.45 (0.10)	1.45 (0.08)	0.50
Serum ferritin (μg/L) [a]	95.9 (2.4)	42.5 (2.8)	<0.01
Alcohol drinker, n (%) [b]	2397 (77.7)	1716 (38.4)	<0.01
Smoker, n (%) [b]	738 (23.9)	218 (4.8)	<0.01
Use of NSAIDs, n (%) [b]	183 (5.9)	228 (5.1)	0.12

OBS: oxidative balance score; PUFA: polyunsaturated fatty acids; SFA: saturated fatty acids; NSAIDs: non-steroidal anti-inflammatory drugs.

[a] Mean (standard deviation); comparisons based on *t*-tests.

[b] Number (%); comparisons based on chi-squared test.

Supplemental Table S3. Results of multiple regression analysis to assess the association between OBS component individual factors or total OBS and log-transformed urinary 8-OHdG/creatinine levels in Japanese men and women

	Men (n=3083) [a]				Women (n=4469) [b]			
	Standardized β	β	95% CI	*P*	Standardized β	β	95% CI	*P*
Total OBS with vitamin D	-0.15	-0.027	(-0.03, -0.02)	<0.01	-0.12	-0.025	(-0.03, -0.02)	<0.01
Total OBS with folic acid	-0.13	-0.022	(-0.03, -0.02)	<0.01	-0.11	-0.135	(-0.03, -0.02)	<0.01
Total OBS created with n-3 PUFA as a component of antioxidant factors	-0.14	-0.025	(-0.03, -0.02)	<0.01	-0.12	-0.023	(-0.03, -0.02)	<0.01

OBS: oxidative balance score; 8-OHdG: 8-hydroxydeoxyguanosine; Standardized β: standardized regression coefficient; β: regression coefficient; CI: confidence interval; PUFA: polyunsaturated fatty acids.

[a] Adjusted for age (years, continuous), total energy intake (kcal, continuous), body mass index (kg/m^2, continuous), hypertension (category), diabetes mellitus (category), and dyslipidemia (category).

[b] Adjusted for age (years, continuous), total energy intake (kcal, continuous), hypertension (category), diabetes mellitus (category), dyslipidemia (category), menopausal status (in women only, category), and body mass index.

Supplemental Table S4. Adjusted geometric means (95% CI) of urinary 8-OHdG/creatinine levels according to equal interval categories of OBS by sex

		OBS				
		Interval 1 (3–6)	Interval 2 (7–10)	Interval 3 (11–14)	Interval 4 (15–18)	*P* for trend [a]
Men	n (%)	501 (16.2)	816 (26.4)	950 (30.8)	819 (26.5)	
	Model 1[b]	4.08 (3.84–4.33)	3.82 (3.73–3.91)	3.45 (3.36–3.54)	3.17 (2.92–3.45)	<0.01
	Model 2[c]	4.09 (3.86–4.34)	3.82 (3.74–3.91)	3.44 (3.35–3.53)	3.15 (2.89–3.42)	<0.01
	Model 3[d]	4.09 (3.85–4.34)	3.83 (3.75–3.92)	3.43 (3.34–3.53)	3.13 (2.88–3.41)	<0.01
Women	n (%)	873 (19.5)	1312 (29.3)	776 (17.4)	1511 (33.8)	
	Model 1	4.45 (4.15–4.78)	4.35 (4.26–4.45)	3.96 (3.86–4.05)	3.60 (3.44–3.78)	<0.01
	Model 2	4.34 (4.06–4.63)	4.34 (4.25–4.43)	3.98 (3.90–4.06)	3.62 (3.46–3.78)	<0.01
	Model 3	4.35 (4.08–4.64)	4.34 (4.26–4.43)	3.97 (3.89–4.06)	3.61 (3.45–3.77)	<0.01

OBS: oxidative balance score; CI: confidence interval; 8-OHdG: 8-hydroxydeoxyguanosine.

[a] Trend tests were performed by including the ordinal numbers 0 to 3 assigned to each quartile category of the OBS in a multiple linear regression analysis.

[b] Unadjusted.

[c] Adjusted for age (years, continuous), total energy intake (kcal, continuous), hypertension (category), diabetes mellitus (category), dyslipidemia (category), and menopausal status (in women only, category).

[d] Adjusted for age (years, continuous), total energy intake (kcal, continuous), hypertension (category), diabetes mellitus (category), dyslipidemia (category), menopausal status (in women only, category), and body mass index (kg/m^2, continuous).
